# Clinical management and outcome of adult patients with extracorporeal life support device–associated intracerebral hemorrhage—a neurocritical perspective and grading

**DOI:** 10.1007/s10143-020-01471-4

**Published:** 2021-01-23

**Authors:** Vincent Prinz, Lisa Manekeller, Mario Menk, Nils Hecht, Steffen Weber-Carstens, Peter Vajkoczy, Tobias Finger

**Affiliations:** 1 Department of Neurosurgery, Charité-Universitätsmedizin Berlin, Charitéplatz 1, 10117 Germany; 2 Department of Anesthesiology and Operative Intensive Care Medicine, Campus Virchow-Klinikum, Charité - Universitätsmedizin Berlin, corporate member of Freie Universität Berlin, Humboldt-Universität zu Berlin, and Berlin Institute of Health, Berlin, Germany

**Keywords:** Extracorporeal membrane oxygenation, ECMO, Intracerebral hemorrhage, Classification, Surgery, Outcome, Coagulation

## Abstract

Intracerebral hemorrhage (ICH) is a devastating complication in patients treated with extracorporeal membrane oxygenation (ECMO) due to respiratory or cardiac issues. Neurosurgical evaluation and management of such cases has only insufficiently been studied. We conducted a retrospective, cohort study of adult patients treated with ECMO between January 2007 and January 2017 in a tertiary healthcare center. Demographics, clinical data, coagulation status, ICH characteristics, and treatment modalities were analyzed. The primary outcome parameter was defined as mortality caused by ICH during ECMO. 525 patients with ECMO therapy were eligible for analysis. An overall incidence for any type of intracranial bleeding of 12.3% was found. Small hemorrhages accounted for 6.4% and acute subdural and epidural hematoma for 1.2%. Twenty-four (4.6%) patients developed ICH, and 11 patients (46%) died due to the ICH. Mortality was significantly higher in patients with larger ICH volumes (86.8 ± 34.8 ml vs 9.9 ± 20.3 ml, *p* < 0.001), intraventricular hemorrhage (83% vs 8%, *p* = 0.01), and a fluid level inside the ICH (75% vs 31%, *p* = 0.04). All patients were classified according to the bleeding pattern on the initial CT scan into 3 types. Patients with type 1 bleeding were statistically more likely to die (*p* < 0.001). In 15 out of 24 patients (63%), correction of the coagulation status was possible within 12 h after ICH onset. Seven out of 9 patients (78%) without early coagulation correction died compared to 2 out of 15 patients (13%), in whom early coagulation correction was successful (*p* = 0.01). This is the first study evaluating the course and management of patients experiencing an ICH under ECMO therapy and establishing an ICH classification based on the bleeding patterns. Early correction of the coagulation is of paramount importance in the treatment of these patients.

## Introduction

Extracorporeal life support devices, especially extracorporeal membrane oxygenation techniques (ECMO), have become well-established methods in intensive care medicine for the treatment of severe respiratory and cardiorespiratory failure [13]. Initially invented in the 1970s, nowadays, the use of extracorporeal heart-and-lung replacement methods has almost doubled in the past decade [27]. The data from a randomized controlled trial as well as an observational study have reported favorable outcomes in patients treated with ECMO while suffering acute respiratory distress syndrome (ARDS) associated with the 2009 influenza epidemic [1]. In the future, the use of ECMO is expected to further increase [6, 20, 25, 28].

Current WHO interim guidelines recommend that patients with ARDS and refractory hypoxemia should be transferred to specialized centers capable of providing ECMO support [20]. However, despite the technological advances over the last decades, even in experienced centers, these procedures are associated with relevant complications like thrombosis, embolism, infection, and an increased risk of bleeding [4]. Intracerebral hemorrhage (ICH) is a severe complication and has been reported as the leading cause of death for veno-venous (vv) as well as veno-arterial (va) ECMO [3, 10]. The difficulties are that during early ECMO therapy, patients are usually sedated and neurologically not assessable. Second, standardized screening protocols and classification systems for the type of intracranial bleeding do not exist, considering a variable ICH incidence between 1.8 and 21% that likely remains underreported [3, 19, 27]. Against this background, a recent systematic review has suggested mortality rates in ECMO-associated ICH cohorts ranging from 32 to 100% [10]. In such a setting, acute neurosurgical evaluation is often required but despite its high relevance and impact on morbidity and mortality, however, detailed information on neurosurgical evaluation and management of ECMO-associated ICH has not yet been systematically assessed. Most importantly, the different types of intracranial bleeding are usually neither distinguished nor stratified, although the pathophysiology and treatment concepts for different types of intracranial bleedings such as parenchymal ICH, non-aneurysmatic subarachnoid hemorrhage (SAH), and also subdural or epidural hematoma (SDH, EDH) remain fundamentally distinct [5, 24, 26]. Here, we studied the relationship between ECMO-associated ICH, bleeding patterns, neurosurgical management, and clinical outcome.

## Materials and methods

### Study population

This retrospective cohort study was conducted in a tertiary academic healthcare center. All consecutive patients with pulmonary or cardiac failure and ECMO therapy (vv-ECMO or va-ECMO) between January 2007 and January 2017 were screened. Criteria for inclusion into this study were as follows: age ≥ 18 years, treatment with vv-ECMO or va-ECMO, and development of an ICH under therapy. For the sake of simplicity, the use of the additions “va” and “vv” with regard to the type of ECMO therapy will be omitted in the further course of the manuscript. “ECMO” includes both types of therapy.

**Fig. 1:**
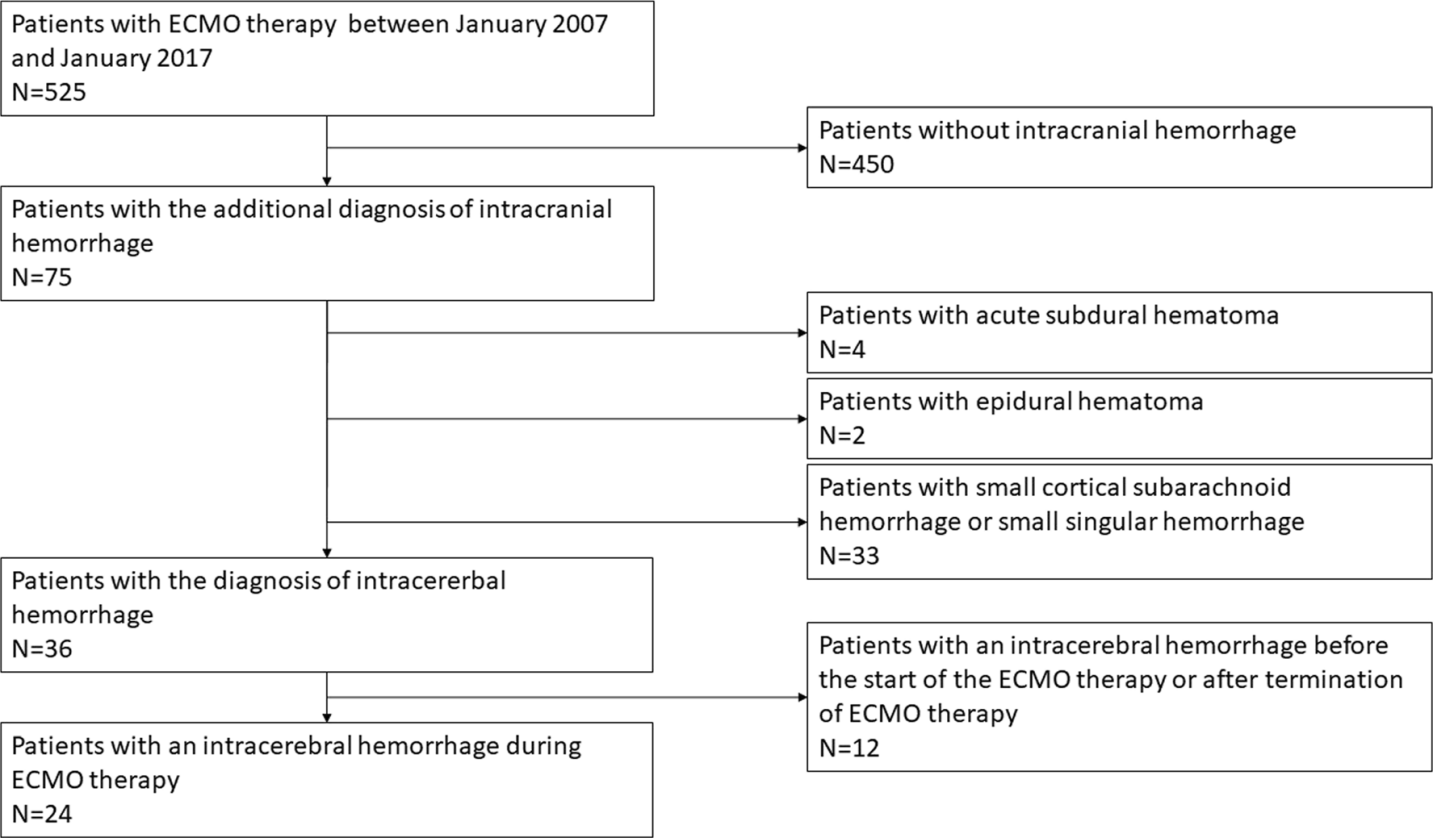
Patient flow chart showing inclusion/exclusion process

As it is a standard operating procedure in our institution, all patients were evaluated with a cranial CT scan before the start of ECMO therapy. All patients with intracranial bleeding before the start of the ECMO therapy and all patients who developed intracranial bleeding after the end of the ECMO therapy were excluded. During ECMO therapy, neurologic examinations were performed 8-hourly by the treating intensive care physician and 4-hourly by the nurse in charge. A follow-up cranial CT scan was initiated if a new neurologic symptom was found like anisocoria, mydriasis, seizures, or persistent coma on sedation withdrawal*.* Patients with small singular cerebral hemorrhages (diameter < 5 mm) or small cortical subarachnoid hemorrhages, as well as subdural or epidural hematoma, were included in the first step of our statistical analysis (Fig. 1, Table 1). The detailed analysis focused on patients with large ICHs. Those patients were divided into two groups: group 1 are patients surviving the ICH and group 2 are patients who died due to the ICH. During the clinical follow-up, patients were repeatedly evaluated with cranial CT scans.

**Table 1:** Overall incidence and mortality of ECMO-associated intracranial bleedings

Pathology	Incidence, % (*n* patients/*n* total patients)	Mortality due to the hemorrhage, % (*n* patients/*n* total patients)	Overall mortality, % (*n* patients/*n* total patients)	*p* value vs no hemorrhage
No hemorrhage	87.7% (450/513)	n/a	46% (207/450)	n/a
Singular small hemorrhage	6.4% (33/513)	0% (0/33)	48.5% (16/33)	0.783
Large hemorrhage	4.7% (24/513)	45.8% (11/24)	79.2% (19/24)	0.001
Acute subdural hematoma	0.8% (4/513)	0% (0/4)	100% (4/4)	0.001
Epidural hematoma	0.4% (2/513)	0% (0/2)	100% (2/2)	0.001
All hemorrhages combined	12.3% (63/513)	17.5% (11/63)	65.1% (41/63)	0.004

All patients with an intracranial hemorrhage before the start of the ECMO therapy or after termination of ECMO therapy (*n* = 12) were not included in this analysis

### Data collection

Patient data was collected by chart review approved by the local ethics committee of the Charité-Universitätsmedizin Berlin (reference number: EA1/007/19). The following clinical variables were extracted: age, gender, cause of ARDS or cardiac failure, duration of ECMO therapy until bleeding onset, last coagulation status before the time point of ICH onset, time until normalization of the coagulation (< 12 h vs > 12 h) after the ICH diagnosis (normalization was defined as INR < 1.25, PTT < 46 s, thrombocytes > 100/nl) [12], number and type of neurosurgical interventions and mortality caused by the ICH.

### Imaging studies

All patients were evaluated with a standard CT scan and the possibility of three-dimensional image reconstruction and analysis. The volume of the ICH, location, and the presence of an intraventricular hemorrhage (IVH) were determined.

In addition, patients were distinguished according to the presence of single or multiple hemorrhages at the time point of ICH diagnosis. The presence of a fluid level as a potential indicator for severe local coagulation impairment was documented as well [11]. In a final step, we defined three distinct ICH types that we observed during ECMO therapy.

The ICH volume was evaluated with the software “iPlan Cranial 3.0” (Brainlab™) on the first CT scan that documented the hemorrhage and volumetric analysis was performed by three independent researchers; the mean value was used for statistical analysis. In patients with more than one hemorrhage location, the cumulative volume of all hemorrhages was used for analysis. All follow-up CT scans were screened for ischemic cerebrovascular complications.

### Coagulation management

Until the onset of the ICH, all patients under ECMO therapy were routinely treated with anticoagulants and received continuous argatroban or unfractionated heparin. The coagulation status during ECMO therapy was monitored at bedside with coagulation tests every 8 h. If a stable coagulation was observed during the course of treatment, the examination intervals were extended to once a day. The partial thromboplastin time (norm: 30.0–46.0 s) was maintained between 50 and 70 s and the thrombocyte count (norm: 240–520/nl) above 50/nl [21]. After ICH diagnosis, anticoagulation was stopped and patients were substituted with prothrombin complex concentrate (PCC) targeting a complete normalization of the patient’s coagulation.

### Statistical analysis

ANOVA was used to test differences in continuous variables and the *χ*^2^ test for contingency analysis. Multivariate analysis was performed with one-way MANOVA for the following variables: ICH volume, duration until normalization of the coagulopathy, presence of ventricular hemorrhage, larger surgical interventions, and a fluid level inside the ICH. Partial Eta-squared values were calculated to estimate the effect size. The interpretation of the values was done in accordance with published recommendations (0.2 small effect size, 0.5 medium effect size, and 0.8 large effect size) [16]. Statistical significance was set at *p* < 0.05. SPSS software (SPSS 21.0) was used for all analyses.

## Results

The initial patient sample consisted of 525 patients being treated with ECMO therapy between January 2007 and January 2017. After applying inclusion and exclusion criteria, the sample size was reduced to 513 patients. In 63 patients, an ECMO-associated intracranial hemorrhage was diagnosed during the hospital admission. The cumulative incidence for the development of ECMO-associated intracranial bleeding in this cohort was 12.3%. Broken down into individual entities, small hemorrhages accounted for 6.4% (33/513), large ICH for 4.7% (24/513), acute SDH for 0.8% (4/513), and EDH for 0.4% (2/513). The overall mortality of all included patients was 48.3% (248/450). There was no significant difference in mortality between patients without a hemorrhage (46%; (207/450)) and patients with a small hemorrhage (48.5%; (16/33); *p* = 0.783). All other types of intracranial bleedings showed a highly significant increase in overall mortality compared to patients without a bleeding event (see Table 1). The six patients who experienced an acute SDH or an EDH did not succumb to the consequences of their bleeding but to their underlying disease (irreversible lung damage (*n* = 3), generalized cerebral hypoxia (*n* = 1), multi-organ failure (*n* = 1), and terminal cardiac insufficiency (*n* = 1)). The only patient group who died due to their cerebral bleeding was the group 11 of the 24 patients with a large ICH. In this patient cohort, the mean age was 49 years ± 16 years with a gender distribution of 54% males and 46% females (see Fig. 1, Table 2).

**Table 2:** Demographic data, information concerning the surgical procedure, the coagulation status, and the ICH data

	All patients (*n* = 24)	Patients surviving the ICH (*n* = 13)	Patients who died because of the ICH (*n* = 11)	*p* value
Age, years (mean ± SD)	48.8 ± 16.1	51.5 ± 17.2	45.6 ± 14.8	0.38
Gender (m/f)	13/11	8/5	5/6	0.45
Duration of ECMO therapy until ICH occurrence (days, mean ± SD)	6.7 ± 7.3	4.5 ± 4.5	9.7 ± 9.2	0.09
Surgical data				
Observation only (*n* cases; %)	17 (63.0%)	10 (66.7%)	7 (58.3%)	0.67
Placement of an ICP probe (*n* cases; %)	3 (11.1%)	3 (20.0%)	0 (0%)	0.11
Placement of an EVD (*n* cases; %)	4 (14.8%)	2 (13.3%)	2 (16.7%)	0.82
Hematoma evacuation (*n* cases; %)	2 (7.4%)	0 (0%)	2 (16.7%)	0.11
Hemicraniectomy (*n* cases; %)	1 (3.7%)	0 (0%)	1 (8.3%)	0.27
Conservative (observation ± ICP ± EVD) vs not conservative (hematoma evacuation ± hemicraniectomy) (*n* patients)	21/3	13/0	8/3	< 0.001
Coagulation status				
Prothrombin time (INR; norm: 0.90–1.25)	1.38 ± 0.21	1.39 ± 0.20	1.36 ± 0.23	0.78
Partial thromboplastin time (seconds; norm: 30.0–46.0)	58.0 ± 13.7	60.9 ± 14.1	54.6 ± 13.0	0.27
Thrombocyte count (per nl; norm: 240–520)	74 ± 56	76 ± 58	71 ± 57	0.85
Fibrinogen (g/l; norm: 1.60–4.00)	3.65 ± 1.57	3.96 ± 1.82	3.23 ± 1.13	0.28
Normalization of the coagulopathy < 12 h after ICH diagnosis possible (yes; %)	15/24; (62.5%)	11/13; (84.6%)	4/11; (36.4%)	0.01
ICH data				
ICH volume (ml)	45.2 ± 47.7	9.9 ± 20.3	86.8 ± 34.8	< 0.001
ICH location				
Frontal (*n* patients; yes/no)	19/5	10/3	9/2	0.78
Temporal (*n* patients; yes/no)	9/15	6/7	3/8	0.36
Parietal (*n* patients; yes/no)	16/8	9/4	7/4	0.78
Occipital (*n* patients; yes/no)	9/ 15	7/6	2/9	0.08
Ventricular hemorrhage (yes/no)	11/13	3/10	8/3	0.01
Singular or multiple hemorrhages (*n* singular/*n* multiple)	9/15	3/10	6/5	0.12
Fluid level in the ICH (yes/no)	8/16	2/11	6/005	0.04

The reasons for ECMO therapy were bacterial pneumonia (*n* = 11), influenza H1N1 pneumonia (*n* = 4), fungal pneumonia (*n* = 3), end-stage cystic fibrosis (*n* = 2), eosinophilic pneumonia (*n* = 1), influenza B pneumonia (*n* = 1), amniotic fluid embolism (*n* = 1), and traumatic lung contusion (*n* = 1). Patient characteristics, surgical data, coagulation characteristics, and ICH data are summarized in Table 2. In our patient population, 11 out of 24 patients (46%) died because of ICH during ECMO therapy, either as a result of increased intracranial pressure with herniation or a withdrawal of care decision considering that the prognosis was incompatible with the patient’s will.

**Fig. 2:**
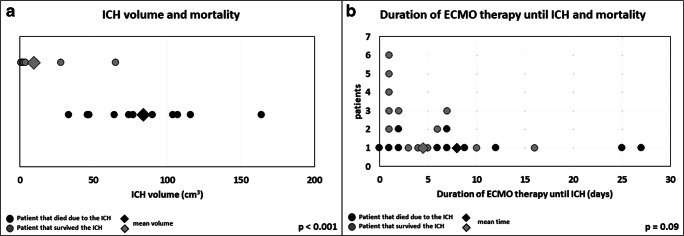
**a** Volume of the ICH in square centimeters of patients under ECMO therapy divided according to the outcome. **b** Total ECMO treatment duration until the onset of the ICH in days. A black dot corresponds to one patient who died and a gray dot to one patient who survived. A black diamond shows the mean value of all patients who died and a gray diamond the mean value of all patients who survived (*N* = 24)

Patients who died because of ICH had a statistically significant larger ICH volume compared to patients who survived with ICH (group 1 = 86.8 ± 34.8 ml, group 2 = 9.9 ± 20.3 ml, *p* < 0.001). After calculation of an odds ratio at an ICH threshold volume of 50 ml, we determined that patients with an ICH volume above 50 ml were 10.3 times more likely to die because of the ICH (Fig. 2).

The duration of ECMO therapy until ICH onset in all patients was 6.5 ± 7.2 days (median: 4.5 days; range: 0–27 days). Between the two groups, there was no significant difference (group 1 = 4.5 ± 4.5 days, median: 3 days, range: 1–16 days; group 2 = 8.8 ± 9.2 days, median: 7 days, range: 0–27 days; *p* = 0.09) (Fig. 2).

**Fig. 3:**
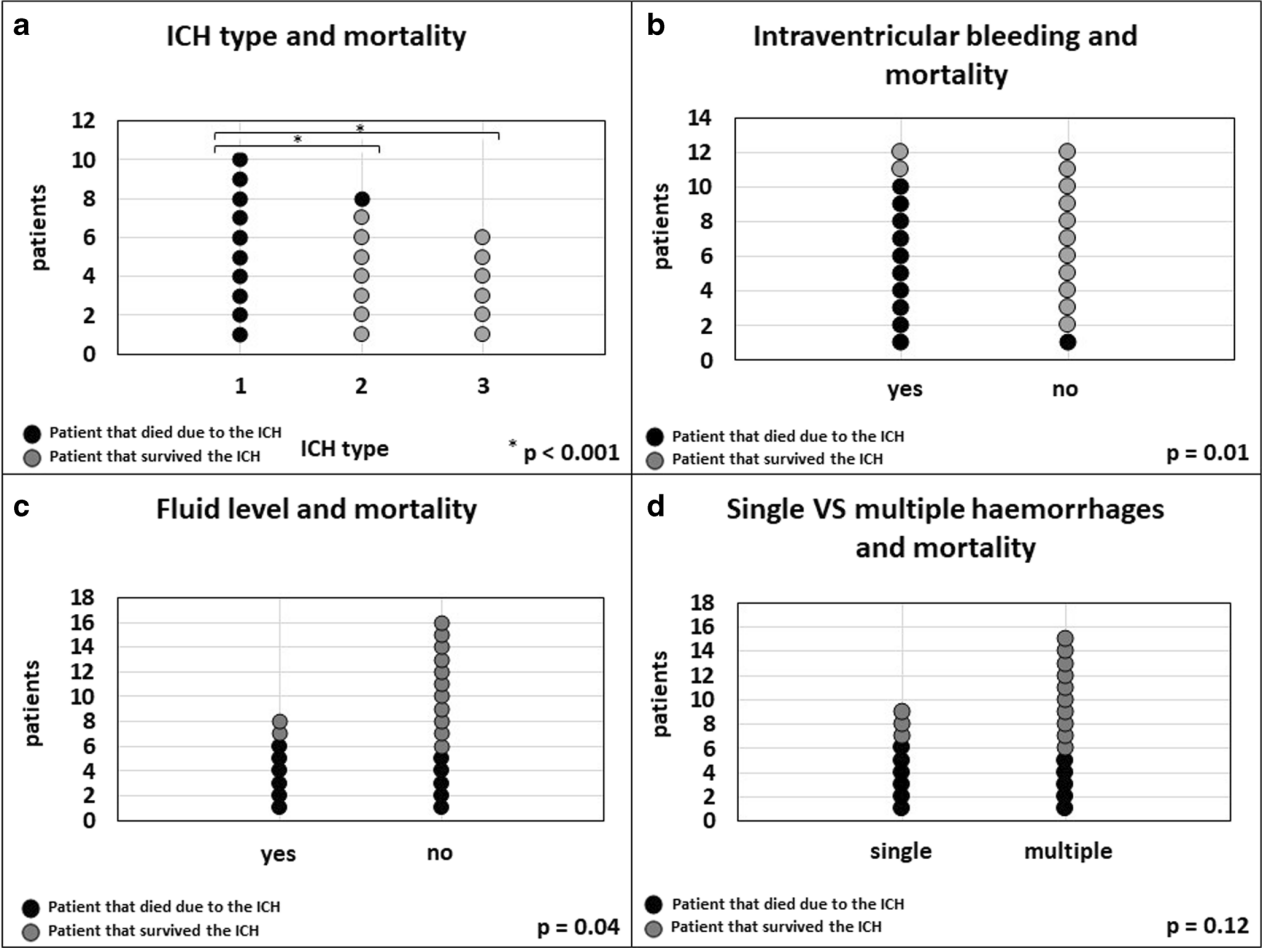
Evaluation of the CCT scan that led to the diagnosis of the ICH. **a** Distribution of all patients according to the bleeding type. **b** Comparison of all patients if an intraventricular hemorrhage was present. **c** Comparison of all patients if a fluid level was present in the hemorrhage. **d** Comparison of all patients with a single hemorrhage to patients with multiple hemorrhages. A black dot corresponds to one patient who died and a gray dot to one patient who survived (*N* = 24)

Presence of IVH (*n* = 12) was associated with a higher mortality (*n* = 10; 83%) compared to patients without IVH (*n* = 1; 8%) (*p* = 0.01; Table 3). In 8 of the 24 patients (33%), we observed a fluid level inside the ICH cavity: The mortality rate in those patients was 75% compared to 31% in patients without an ICH fluid level (*p* = 0.04; Table 2). Interestingly, the presence of multiple hemorrhages had no impact on mortality (singular: 6 out of 9 patients; multiple: 5 out of 15 patients) (*p* = 0.12) (Fig. 3**)**.

**Table 3:** Multivariate analysis (one-way MANOVA) of the evaluated dependent variables, including the estimate of effect size (partial Eta-squared values; Cohen et al. 1988; interpretation of values: 0.2 small effect size, 0.5 medium effect size; 0.8 large effect size) and significances

		Dependent variable				
		ICH volume	Normalization of the coagulopathy < 12 h after ICH diagnosis	Ventricular hemorrhage (IVH)	Conservative vs non-conservative treatment	Blood mirror in the ICH
Contrast estimate		76.895	0.937	− 0.497	0.273	− 0.392
Partial Eta-squared		**0.674**	**0.330**	**0.247**	**0.169**	**0.171**
Std. error		11.413	0.284	0.185	0.129	0.184
Sig.		0.000	0.003	0.014	0.046	0.044
95% confidence interval for difference	Lower bound	53.226	0.347	− 0.880	0.005	− 0.772
	Upper bound	100.564	1.527	− 0.113	0.540	− 0.011

**Fig. 4:**
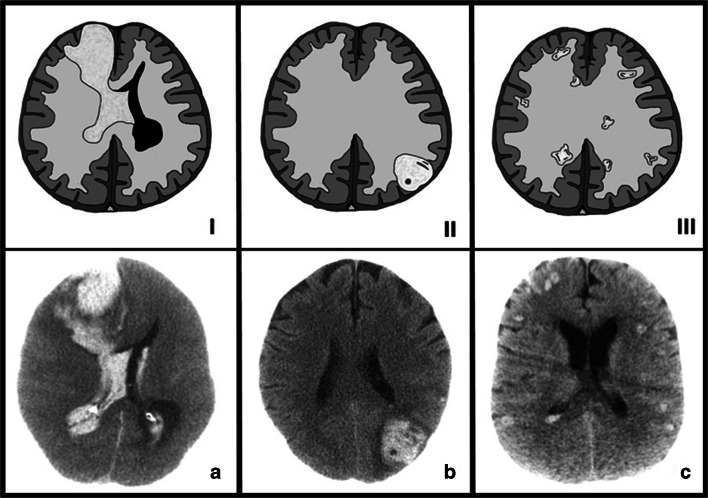
Classification of the different ICH types. Letters I–III showing bleeding schematics and A–C showing clinical examples. Type I/A: lobar bleeding with IVH ± smaller lobar bleedings. Type II/B: one or more lobar bleedings without IVH. Type III/C: multiple (> 3 lesions) small (< 0.5 ml per lesion) broadly scattered lesions ± IVH (*N* = 24)

Based on the ICH distribution, we then categorized patients according to one of the following 3 bleeding types (Fig. 4): type I: lobar bleeding with IVH ± smaller lobar bleedings; type II: one or more lobar bleedings without IVH; type III: multiple (> 3 lesions) small (< 0.5 ml per lesion) and scattered lesions ± IVH. All patients (*n* = 10; 100%) with type 1 bleeding died during the course of treatment compared to 1 out of 8 patients (12.5%) with type 2 bleeding and none of the patients (0%) with type 3 bleeding. Also, patients suffering type 1 bleeding were statistically more likely to die as a result of the ICH itself (*p* < 0.001).

The coagulation status at the time point of ICH onset did not differ between patients who died as a result of the ICH and patients who survived (prothrombin time: 1.39 ± 0.20 vs 1.36 ± 0.23, *p* = 0.78; partial thromboplastin time: 60.9 s ± 14.1 s vs 54.6 s ± 13.0 s, *p* = 0.27; thrombocyte count: 76 ± 58/nl vs 71 ± 57/nl, *p* = 0.85; fibrinogen: 3.96 g/l ± 1.82 g/l vs 3.23 g/l ± 1.13 g/l, *p* = 0.28). However, in 15 out of 24 patients (63%), coagulation was not normalized within the first 12 h after ICH diagnoses and 7 out of 9 patients (78%) without a timely normalization of the coagulation died as a result of the ICH compared to 2 out of 15 patients (13%) in whom normalization of the coagulation succeeded (*p* = 0.01). During follow-up, no evidence of ischemic events was detected on CT imaging.

**Fig. 5:**
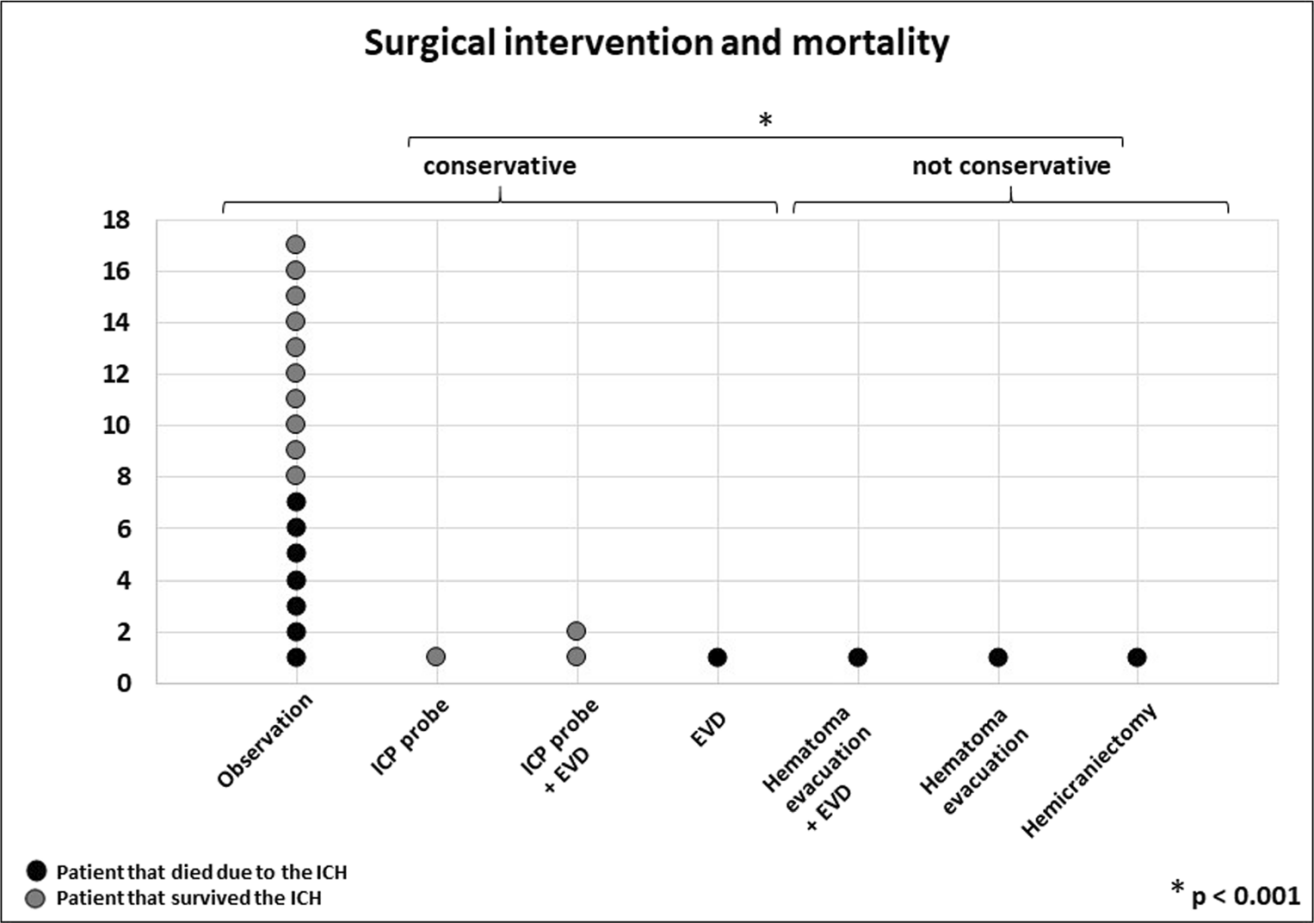
Comparison of patient outcome according to the surgical intervention. For statistical analysis and clinical practicability, we defined observation and the placement of an EVD and/or ICP probe as conservative treatment. A black dot corresponds to one patient who died and a gray dot to one patient who survived (*N* = 24)

After the diagnosis of ICH, 7 patients (29%) were treated surgically and 17 patients (71%) were managed non-surgically. All patients underwent repeated clinical evaluation and CT diagnostics. Surgical interventions included the simultaneous placement of an intracranial pressure (ICP) probe and external ventricular drain (EVD) (*n* = 2), the placement of an ICP probe or an EVD alone (*n* = 2), hematoma evacuation and EVD placement (*n* = 1), hematoma evacuation alone (*n* = 1), and decompressive hemicraniectomy (*n* = 1). Next, we divided patients into a “non-surgical” group, including all patients who were observed ± EVD or ICP placement (*n* = 21) and into a “surgical” group, including all patients with a craniotomy for hematoma evacuation or hemicraniectomy (*n* = 3). All three patients in the “surgical” group died as a result of the ICH (100%), compared to 8 out of 21 patients in the “non-surgical” group (38%) (*p* < 0.001) (Fig. 5).

Multivariate analysis confirmed an independent effect of a larger ICH volume (partial Eta-squared value: 0.674), coagulation in patients that is not normalized within 12 h after ICH onset (partial Eta-squared value: 0.330), presence of an IVH (partial Eta-squared value: 0.247), larger surgical intervention (partial Eta-squared value: 0.169), and a fluid level inside the ICH (partial Eta-squared value: 0.171) towards the likelihood of mortality as a result from the ICH (Table 3).

## Discussion

Our study has the following main findings: (1) the cumulative incidence for ECMO-associated intracranial bleedings in our cohort was 12.3%; (2) no impact of small single or multiple small hemorrhages was found while patients with a large ICH showed a mortality rate caused by the bleeding event of 46%, with a subsequent overall mortality rate of almost 80%; (3) the ICH volume, presence of an IVH, and the ICH type may serve as predictors for mortality; (4) early correction of the coagulation status significantly reduces mortality (78% vs 13%); (5) surgical intervention in ECMO-associated ICH is associated with high mortality rates.

Following the influenza endemic in 2009, ECMO therapy has become an established and important adjunct in intensive care medicine for the treatment of ARDS and severe cardiac failure. However, maintaining ECMO requires a narrow balance between anti- and pro-coagulatory demands. In addition to systemic anticoagulation, ECMO is independently associated with platelet dysfunction [2].

Intracranial hemorrhage is a frequent complication in adult patients treated by ECMO and consequently, neurosurgical evaluation and potential intervention are often requested. So far, evidence-guided decision-making with respect to the type or size of the intracranial bleeding and management of the coagulation status is highly limited. Taking the overall increase of patients requiring extracorporeal life support during the last decade and regionally limited intensive care capacities into account, evidence regarding the management of ECMO-associated intracranial hemorrhages from a neurosurgical perspective is urgently needed [7, 25]. In addition, although the multicenter international extracorporeal life support organization (ELSO) registry and another multicenter database comprise the data of more than 8000 patients, the characteristics and subtypes of intracranial bleedings are neither considered nor reported in detail [18, 22]. So far, data differentiating subtypes of intracranial bleedings are all derived from a few retrospective single-center studies [9, 15, 17]. In these studies, the incidence of intracranial bleeding differed between 16.4 and 21.3%, with ICH being the most frequent intracranial bleeding type. Here, we studied our own ECMO registry, including a total of 513 patients between January 2007 and January 2017. In all evaluated patients, a cranial CT scan was performed before ECMO therapy was initiated. During our study, 5 patients developed an ICH after the ECMO device had been removed. We did not include these patients in the analysis as the earliest time point was 4 days after ECMO treatment was terminated and in all 5 cases, the coagulation status was normalized at the time point of ICH onset.

Overall, we found an in-hospital mortality rate in patients suffering ECMO-associated ICH of 65%, which appears slightly lower considering previous reports of mortality rates around 70% [9, 15, 19]. If only the neurosurgical relevant ICHs are analyzed, the overall mortality rate was as high as 80%. A study from the UK analyzed patients with and without ECMO-associated intracranial hemorrhage and found no relevant difference in 6-month survival (68.3 vs 76%) [17]. This stands in a stark contrast to our survival rate at the time point of discharge (34.9% vs 54%). However, due to the complex and diverse patient population and lack of standardized definitions of intracranial bleedings and screening protocols, a reliable comparison between previous reports and also our present findings naturally remains hampered.

Although a number of parameters (e.g., ICH volume, presence of IVH, level of consciousness at diagnosis, bleeding pattern) and scoring systems to evaluate the prognosis of different types of ICH have been evaluated [8, 14, 23], so far, these have not been applied to ECMO patients. Based on the data from our cohort, we focused on certain ICH types and defined three different bleeding patterns with predictive value regarding mortality. The widely established Hemphill score is not applicable in the setting of ECMO-associated ICH due to mandatory consideration of the initial GCS score, the relatively young age of affected patients, the lack of cerebellar hemorrhage, and the failure to recognize the overall severity of the patients’ illness [14]. In order to facilitate easy and standardized clinical applicability, we deliberately chose a purely morphological score.

Finally, our data highlights the high mortality of ECMO-associated, space-occupying ICH requiring surgical evacuation, because none of the 3 operated patients survived. Of course, these results must be interpreted with caution as only a small number of patients were available for analysis in this subgroup and possibly, the detrimental effect of the surgical procedure is even less than the detrimental effect of the space-occupying hematoma itself. Nevertheless, the unfavorable course of surgically treated patients in our population is in line with the findings of previous studies. A recent review on ECMO-associated ICH reported a mortality of 78% in surgically treated patients [10], including a high selection bias, as, in one study reviewed, more than 40% ECMO therapy was withdrawn after ICH diagnosis [9]. There was no randomization process involved in deciding the coagulation management after ICH diagnosis. Therefore, a valid reliable statement concerning the benefit of an early normalization of the coagulation is not possible with our analysis. One observation, however, was that in patients with a normalization of the coagulation within the first 12 h after diagnosis of the ICH, a significantly lower mortality rate was found. No ischemic events occurred during the follow-up with normalized coagulation. As a limitation, there remains a diagnostic uncertainty as the diagnostic sensitivity of the imaging studies (CT scans) does not allow the detection of small ischemic cerebrovascular events. A timely normalization of the coagulation status additionally improves the safety in the case of implantation of a probe for ICP measurement. In our cohort, all EVDs and ICP probes were placed in a bedside fashion on the ICU reducing further possible complications caused by transportation to the operating room. None of these 4 patients suffered from a postoperative cerebral hemorrhage.

## Conclusion

ICH is a feared and detrimental complication in patients treated with ECMO. As the highest incidence of ICH occurs during the first 7 days after ECMO initiation, frequent routine control scans and a close clinical neurological monitoring are of paramount importance. A swift and not hesitant normalization of the coagulation status may significantly reduce mortality. For all clinical specialties treating these patients, it has to be clear that in the event of an ICH under ECMO therapy, the benefits of normalizing the coagulation seem to outweigh potential prothrombotic complications in the acute phase. To the best of our knowledge, this is the first study that evaluates the course and management of patients experiencing an ICH under ECMO therapy and establishes an ICH classification that has prognostic relevance and is solely based on CT morphological bleeding patterns. Due to the retrospective monocentric nature of our study, the results must be interpreted with caution and need to be confirmed in larger, prospective cohorts. Furthermore, our study is limited, as follow-up clinical outcome data is not available. However, our data provide important insights into management of ECMO-associated ICH under critical clinical conditions. Standardized screening protocols and classification of the type of intracranial bleeding are warranted to be integrated into further ECMO studies and registries.

## Data Availability

Data will not be deposited.
